# Innovative Applications of Laser Technology for Bedridden People Posture Monitoring †

**DOI:** 10.3390/s25072108

**Published:** 2025-03-27

**Authors:** David Sánchez Canzobre, Pablo Pardiñas Torrado, Javier Lamas Vigo, Alberto Ramil Rego

**Affiliations:** Centro de Investigación en Tecnologías Navales e Industriales, Universidade da Coruña, Campus Industrial de Ferrol, 15403 Ferrol, Spainalberto.ramil@udc.es (A.R.R.)

**Keywords:** structured light, disabled people care, laser, diffractive optical element

## Abstract

This article presents an innovative system for the automated classification of postures of bedridden people using laser technology. The proposed method leverages low-cost structured light hardware and a custom algorithm to determine the side on which a person is lying. The solution prioritizes patient privacy by avoiding the capture of visual images while maintaining high accuracy in classifying postures. The system includes an enhanced hardware design with structured infrared light that is tailored for affordability and simplicity, and it incorporates an improved algorithm capable of robust posture detection. The experimental results obtained under real-world conditions validate the system’s performance and highlight its potential for deployment in clinical and home care environments. This work expands upon a previous study presented at the 6th International Conference on Application of Optics and Photonics, offering additional insights, refined methodologies, and a broader scope of validation.

## 1. Introduction

Aging populations pose significant challenges to healthcare systems, particularly in the care of dependent elderly individuals. According to the 2018 Report: Older People in Spain prepared by the Ministry of Health, by 2060, almost half of the elderly population will be octogenarians, significantly increasing the demands on the social and healthcare sectors [1]. In this context, continuous monitoring of bedridden individuals’ postures has been identified as a critical need to prevent associated complications, such as pressure ulcers.

Several approaches have been developed for postural monitoring, primarily relying on pressure sensors, computer vision, and machine learning. For example, Chen et al. conducted an exhaustive review of non-contact sleep monitoring techniques, highlighting methods based on sensors and cameras [2]. Among specific solutions, Matsui et al. proposed an automated system for sleep position classification using a pressure-sensitive bed sheet [3], while Park et al. introduced flexible wearable sensors for posture detection [4]. Although effective, pressure sensor-based approaches have notable limitations in long-term stability due to material fatigue, sensor displacement, and mechanical wear, requiring periodic recalibration. Yang et al. combined pressure sensors with machine learning to improve classification accuracy, but their method was only tested under controlled conditions [5].

In the field of computer vision, Gao et al. employed depth cameras and convolutional neural networks for posture classification [6], while Li et al. investigated infrared thermal imaging for non-contact monitoring [7]. Zhao et al. explored smart bed sheets with integrated sensors for real-time posture classification [8], and Li et al. demonstrated the effectiveness of ultra-wideband radars for non-intrusive monitoring, though their implementation requires specialized hardware [9]. Additionally, Khan et al. developed a system based on wearable sensors and machine learning techniques, emphasizing its applicability in both clinical and home environments [10].

Machine learning and neural networks have also been widely used in this field. Supratak et al. examined deep learning techniques for sleep stage classification, emphasizing the need for large and labeled datasets [11]. Liu et al. explored the use of transfer learning for in-bed posture classification using pressure sensors [12], while Zheng et al. proposed pressure-sensitive mattresses combined with machine learning to generate three-dimensional posture profiles [13].

Depth sensors have also been applied for non-intrusive sleep monitoring, as was demonstrated by Sun et al., who developed a depth-sensing system to analyze postural positions [14]. Flexible wearable sensors have also proven effective for real-time monitoring [4]. However, many of these solutions suffer from high hardware costs, complex maintenance requirements, and the need for extensive training datasets, limiting their practical scalability in clinical settings.

In contrast, our approach utilizes a low-cost structured infrared light system that overcomes many of these challenges. Unlike pressure sensors, which degrade over time and require frequent recalibration, our method provides long-term stability by eliminating physical contact with the patient. Additionally, our system does not require stereo cameras, depth sensors, or machine learning, instead relying on traditional algorithms to analyze the deformation of the projected structured light pattern. This significantly simplifies both implementation and maintenance, making it an ideal solution for nursing homes and clinical environments where reliability, ease of use, and affordability are critical. Furthermore, our approach prioritizes patient privacy by avoiding the capture of visual images, addressing ethical concerns often associated with video-based monitoring systems.

This article builds upon our previous work, Use of Laser Technology for the Postural Classification of Bedridden People [15], in which we introduced a preliminary structured light system. In this extended version, we describe the improvements in system design, detail the additional tests conducted under real-world conditions, and provide a more comprehensive analysis of the obtained results.

Following this introduction, the document provides a detailed overview of the project’s development. The algorithm that was designed to determine the posture of a bedridden person is presented, followed by the results obtained from initial tests. Subsequently, an exhaustive description of the hardware is provided, along with an analysis of the tests conducted to verify the viability of the proposed solution. This article concludes with a discussion of the inferences drawn from the findings, offering a comprehensive perspective on the project’s progress and future implications.

## 2. Posture Classification Algorithm

In clinical and elderly care environments, bedridden patients who are unable to move without assistance are frequently placed in a safe position, also known as the lateral decubitus position. To prevent the development of pressure ulcers and other skin injuries, healthcare professionals must periodically reposition these patients. Failing to do so can lead to serious medical complications.

In this initial stage of development, our algorithm is specifically designed to address the following particular use case: detecting whether a patient is lying on their left or right side in a lateral decubitus position. By continuously monitoring the posture, the system can generate alerts for caregivers when a patient has remained in the same position beyond the recommended time threshold, helping to ensure timely repositioning and reducing the risk of pressure sores.

Currently, the use of artificial intelligence (AI) for image classification is a growing trend. However, one of the main drawbacks of these technologies is the need for a large number of labeled images to train AI models. In our case, due to lacking a set of labeled images and considering the significant effort it would take to create one, we opted to develop an algorithmic solution.

The proposed algorithm, whose pseudocode is shown in Appendix A, was implemented in Python and determines the position of a bedridden person by analyzing the concavity or convexity of a patient’s leg profile under the sheets. This method is based on identifying the maximum points of the surface created by the body on the bed. From these points, a second-degree polynomial fitting is calculated using the polyfit method from the numpy library. Subsequently, the second derivative criterion is applied to determine the concavity or convexity of this polynomial, which allows us to establish whether the person is lying on their left or right side.

**Figure 1:**
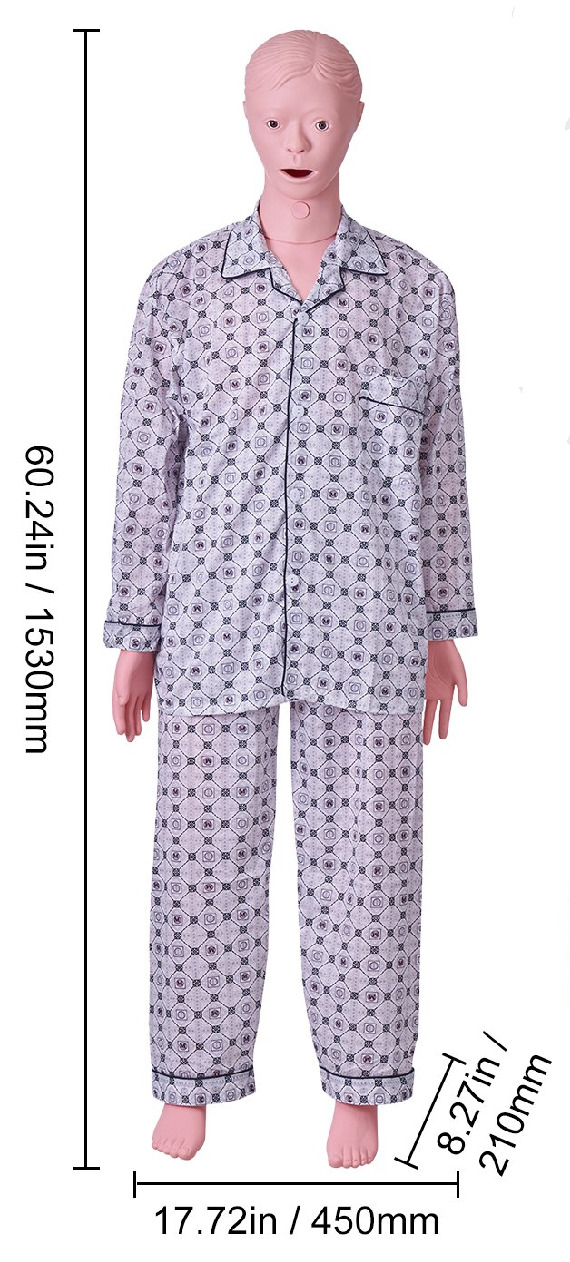
Photograph of the mannequin used during the tests.

**Figure 2:**
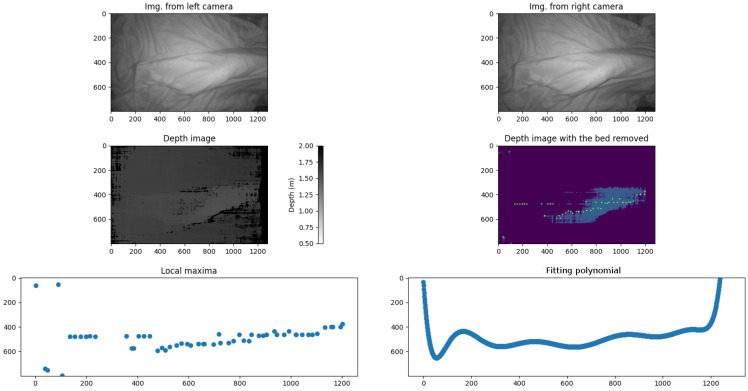
The first row displays the scene captured by both lenses of the stereo camera, showing a person lying on their left side. The second row presents the corresponding depth image, where each pixel shows the distance to the surface, followed by the processed depth image with the bed points removed, isolating only the person’s surface. The third row highlights key analytical steps: the first image marks the local maxima of the extracted profile, while the second image illustrates the fitted polynomial curve, which was computed after outlier removal and evaluated over the range of 0 to 1280 pixels on a horizontal axis. All of the graph scales are expressed in pixels.

**Figure 3:**
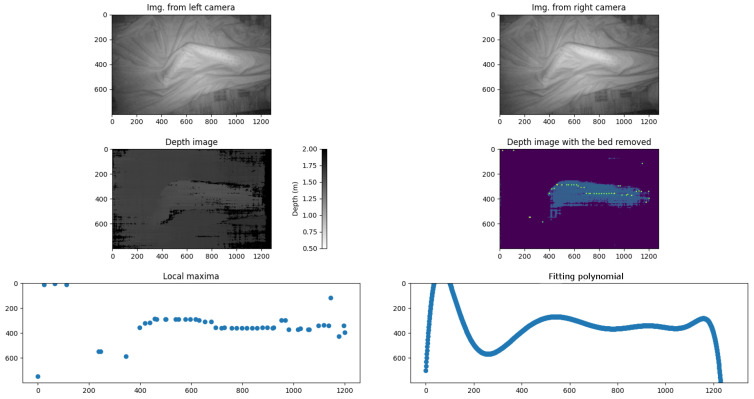
The first row displays the scene captured by both lenses of the stereo camera, showing a person lying on their right side. The second row presents the corresponding depth image, where each pixel shows the distance to the surface, followed by the processed depth image with bed points removed, isolating only the person’s surface. The third row highlights key analytical steps: the first image marks the local maxima of the extracted profile, while the second image illustrates the fitted polynomial curve, which was computed after outlier removal and evaluated over the range of 0 to 1280 pixels on a horizontal axis. All of the graph scales are expressed in pixels.

To validate this approach, a testing phase was conducted using a commercial stereo camera, specifically the Luxonis OAK-D Pro (Luxonis, Colorado, CO, USA). The initial results were promising. All of the tests were performed by scanning the legs of a nursing mannequin, which measured 1.53 m in height (Figure 1). At this stage of the project, no tests were conducted with real subjects. Figure 2 and Figure 3 clearly illustrate the changes in the convexity of the fitting polynomial for the maximum points of the 3D surface, thereby representing the leg profile. The analysis of these convexity changes allowed the algorithm to accurately identify the lateral position of the bedridden person.

## 3. Hardware

Commercial cameras capable of capturing 3D surfaces have a high cost, which makes their deployment challenging in environments such as nursing homes with multiple rooms. An example of this is the OAK-D Pro camera, which was used to validate our algorithm and was priced at around EUR 390 (approximately USD 420) at the time of writing this document. Due to this high cost, and with the aim of avoiding dependence on a single provider (vendor lock-in), we decided to develop our own low-cost structured light system. This solution allowed us to implement affordable technology tailored to our specific needs.

To determine the most suitable structured light system for scanning the surface of a bedridden person, we conducted a comprehensive review of theexisting literature, with a particular focus on the works of J. Salví et al. Our research centered on two key publications: “Pattern Codification Strategies in Structured Light Systems” [16] and “A State of the Art in Structured Light Patterns for Surface Profilometry” [17]. These studies provided an extensive overview of the various structured light techniques and their applications, guiding our evaluation of the most appropriate options for our specific use case. This thorough examination of the current research enabled us to make informed decisions regarding the optimal structured light system for our project’s unique requirements.

### 3.1. Background and Rationale for Hardware Selection

Given the constraints of the clinical environment—where it is crucial not to disturb the patient and to keep costs as low as possible—we opted for a structured light system based on infrared laser technology. Our decision was heavily influenced by the need to balance effectiveness, patient comfort, and cost-efficiency, while also addressing the unique challenges of clinical settings.

Infrared lasers offer several advantages that make them particularly well suited for this application. Minimizing patient disturbance was a primary concern as monitoring systems in hospitals and nursing homes must function without disrupting the patient’s rest or daily activities. Since infrared light is invisible, it allows for continuous, non-intrusive monitoring, making it more practical than structured light systems operating in the visible spectrum. Additionally, infrared projection remains stable under variable clinical lighting conditions unlike traditional structured light systems, which can be affected by ambient illumination changes.

Patient privacy was also a key factor in our choice of infrared laser-based structured light over standard camera-based vision systems. By avoiding the capture of identifiable visual information, our system complies with privacy regulations while maintaining accurate posture classification.

From a cost and maintenance perspective, infrared lasers provide a low-cost and durable solution, reducing the need for frequent recalibrations compared to depth cameras or pressure sensor-based systems. The use of a fixed laser-camera setup ensures long-term monitoring stability without the degradation issues commonly associated with contact-based solutions. Additionally, the non-contact nature of the system allows it to seamlessly integrate into clinical workflows, requiring minimal intervention from healthcare staff.

Our approach was inspired by the method described in the study Laser Multiple Line Triangulation System for Real-Time 3-D Monitoring of Chest Wall During Breathing [18] by Raiola et al. In their work, a laser and camera setup was used to monitor the chest wall of a patient in real time to assess breathing patterns. Similarly, we employed a comparable configuration for our system, positioning the setup above the patient’s bed to capture the profile of the legs under the sheets. This technique enables the extraction of detailed and accurate surface profiles, which are critical for effective patient assessment.

### 3.2. Choice of Components for the System

For the prototype, we selected an infrared laser to ensure that the system remains non-intrusive and comfortable for the patient. However, during the initial proof-of-concept tests conducted in the laboratory, we used a red light-emitting laser (Picotronic DOE256-650-5-3), Picotronic, Unterhaching, Germany instead. This choice was made due to its visibility, which facilitated system alignment and calibration during early-stage testing. In addition to the laser, two diffractive optical elements (DOEs) were employed to project different structured light patterns: the Picotronic DOE-DE-R284, which creates a parallel line pattern; and the Picotronic DOE-DE-R354, which generates a grid pattern. These optical elements were used to explore different projection strategies and to identify the most effective method for capturing detailed surface profiles.

Similarly, for these preliminary tests, we used the KUROKESU C1 PRO X10 camera (KUROKESU, Tartu, Estonia), as it was the one available in our laboratory. The goal at this stage was to validate whether the structured light system, composed of a laser and a camera, was capable of accurately measuring the patient’s posture before investing in additional hardware. Once the laboratory tests confirmed the system’s measurement capabilities, we proceeded with the acquisition of the Dahua IPC-HDW2231T-AS-S2 (Dahua Technology, Hangzhou, China) camera for the prototype. This model was chosen because it is the most widely used for video surveillance in Raiola’s nursing homes, ensuring seamless integration and compatibility within the target environment. Table 1 and Table 2 show the main characteristics of both video cameras.

The total hardware cost amounted to EUR 319.3, with the price breakdown detailed in Table 3; however, the cost could be significantly reduced using a different camera model. Nevertheless, the cost of the complete system remains lower than that of the stereo camera used for testing the algorithm presented in Section 2, with the retail price of the Luxonis OAK-D Pro being EUR 404.93. Prices were consulted in [21,22,23,24].

### 3.3. Testing and Configuration

To evaluate the measurement capability of the laser and camera assembly, we conducted a series of tests in the laboratory before deploying the setup in a real-world scenario.

#### 3.3.1. Laboratory Testing

During the development of the system for assessing health status based on pose and displacement control, laboratory tests were conducted to verify the functionality and feasibility of the components used. These tests allowed for the adjustment of technical parameters and the determination of the system’s capabilities in controlled environments. The main procedures and results obtained are described below.

The experimental system consisted of several key elements. The equipment included a diode laser model, Picotronic 256-650-5-3, with a power of 5 mW and a wavelength of 650 nm, and it is used to project patterns onto surfaces. Additionally, three diffractive elements were employed: the Picotronic DOE-DE-R284, which generates a pattern of 41 lines; the Picotronic DOE-DE-R354, with a design of 10 × 10 lines; and the Picotronic DOE-DE-R241, which projects a pattern of 21 × 21 points. Image capture was performed using a digital camera model KUROKESU C1 PROL-X10, which was available in the laboratory at the time of testing and differed from the model intended for the final prototype. Complementing these elements, a power supply, optical mounting components to ensure proper device arrangement, and a laptop computer were used to facilitate the data acquisition and analysis during the tests.

**Figure 4:**
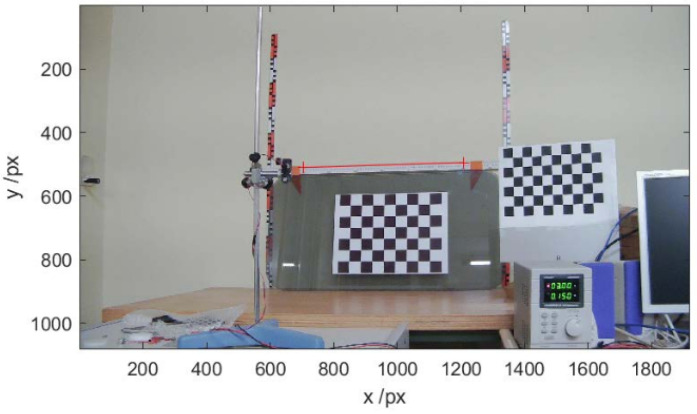
Mounting for system calibration.

In a preliminary stage, the camera was calibrated to determine the system’s essential optical parameters. This process was performed using the camera calibration toolbox provided by MATLAB, which implements Zhang’s method [25] for camera calibration. This algorithm estimates intrinsic parameters such as focal length, principal point, and lens distortion through using multiple images of a known pattern, typically a checkerboard. The calibration procedure involved capturing images of graduated rulers (Figure 4) to measure the distance between the camera and the reference surface. Through this process, a scale of 1.19 mm per pixel, a tilt of −1.82 degrees, a field of view of 59.02 degrees, and a focal distance of 5.63 mm were obtained.

**Figure 5:**
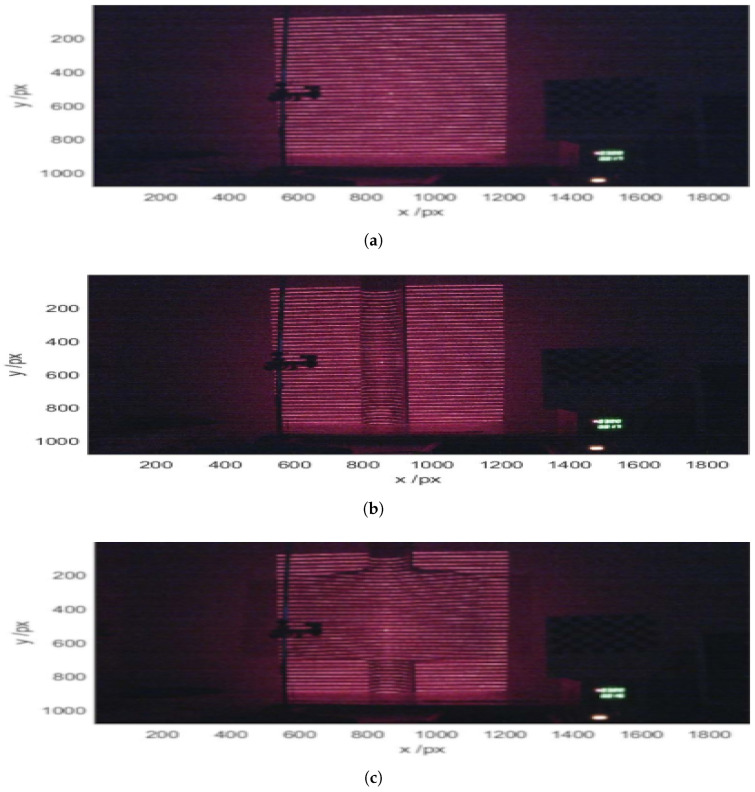
(**a**) Reference image. (**b**) Cylinder without “blanket”. (**c**) Cylinder under a sheet of paper as the blanket.

**Figure 6:**
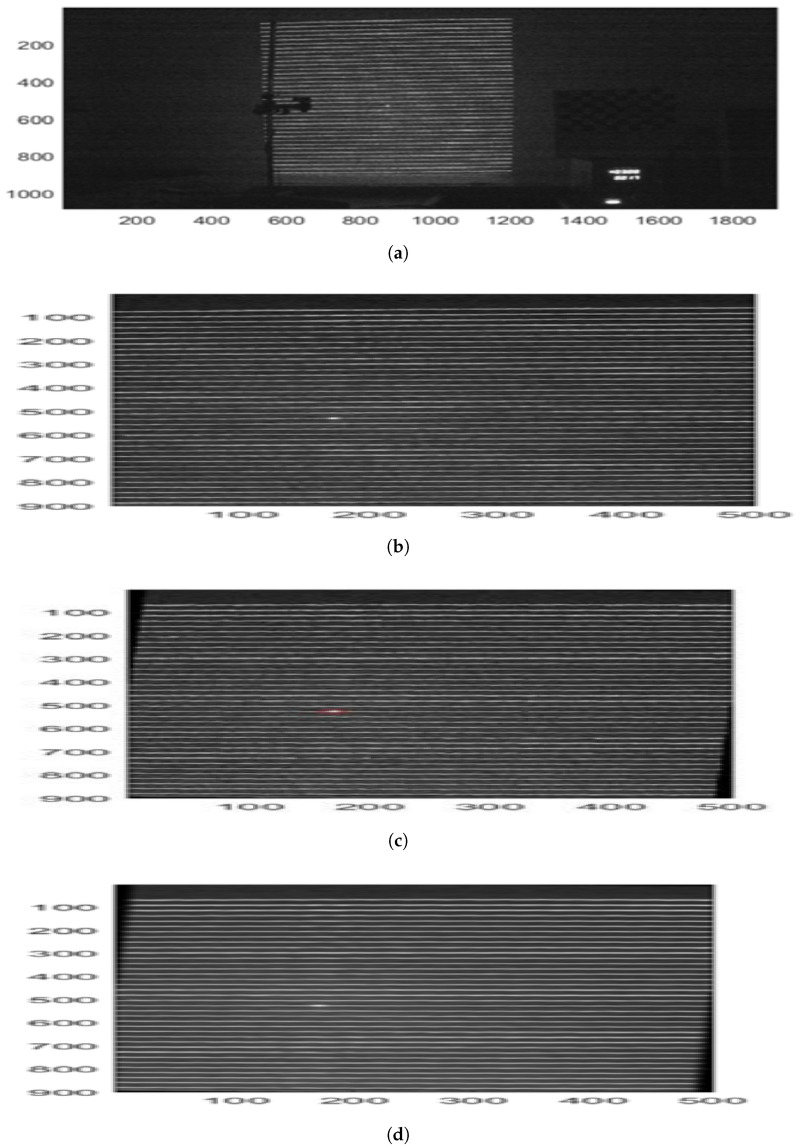

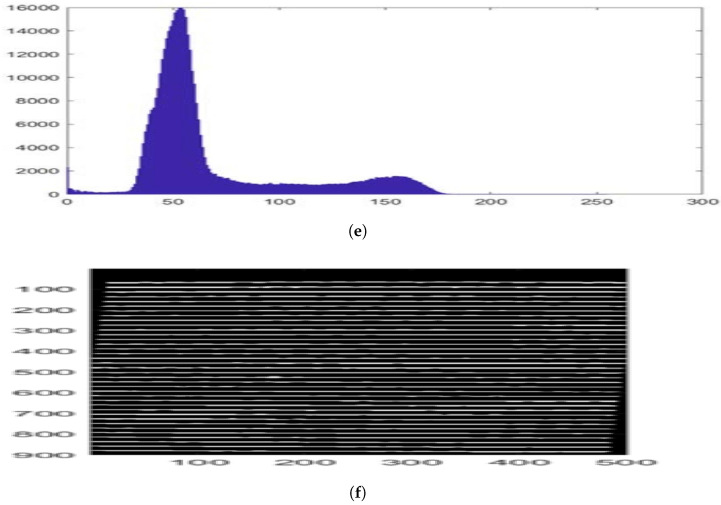
(**a**) Original image. (**b**) Cropped image. (**c**) Lines adjusted to a corrected tilt of −1.86 degrees. (**d**) Gaussian filter applied. (**e**) Line histogram. (**f**) Thresholded image.

The diode laser, coupled with the diffractive elements, was used to project patterns of lines and points onto various surfaces. First, a flat surface (Figure 5a) was used as an initial reference to establish the baseline analysis values. Subsequently, the pattern was tested on a cylinder (Figure 5b), where the deformation due to curvature was analyzed. Finally, the pattern was projected onto a cylinder covered with paper (Figure 5c), allowing for the evaluation of changes induced by surface modification. The images obtained by the camera were processed following a systematic set of steps. First, the images were converted to grayscale (Figure 6a), which were then subsequently cropped (Figure 6b) and aligned, correcting a tilt of −1.86 degrees (Figure 6c). Additionally, a Gaussian filter was applied to smooth the visual information (Figure 6d), followed by a histogram analysis (Figure 6e) that was performed to determine the threshold level for binarization (Figure 6f). Since the optimal threshold can vary depending on lighting conditions and surface reflectance, an adaptive approach was used. The threshold level was automatically determined using Otsu’s method [26], which selects the value that minimizes the intra-class variance in the histogram distribution. This ensured a robust segmentation of the projected structured light pattern across different test conditions.

**Figure 7:**
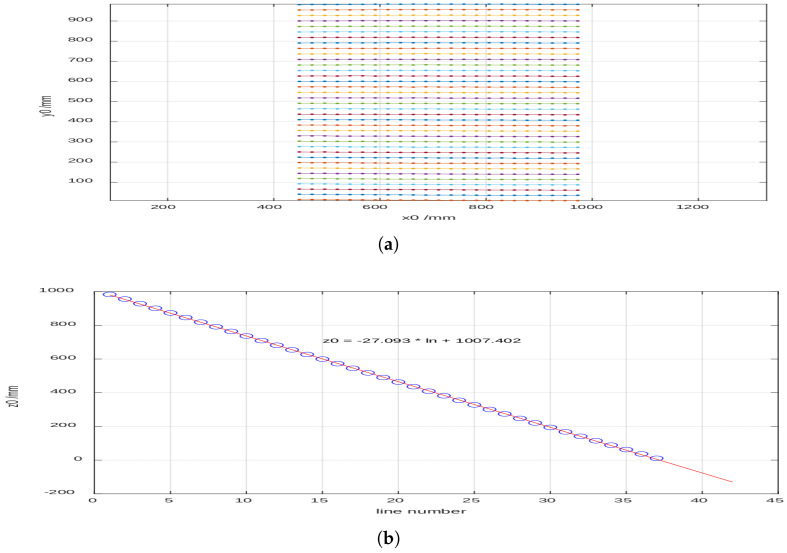
(**a**) The identified positions of the projected pattern lines. (**b**) The linear relationship between the height z and the number of projected line.

Once the binarized images were obtained, the positions of the lines in the projected pattern were identified (Figure 7a). In the case of the flat surface, 37 lines were detected, while, on the cylinder, the number increased to 39 due to curvature. The coordinates obtained in pixels were transformed into real units in millimeters using the camera calibration parameters. The analysis enabled the determination of relative heights based on the linear relationship between height z and the number of projected lines (Figure 7b). For non-flat surfaces, the vertical displacement of each line was calculated, and, combining these data with the horizontal and vertical displacement ratio, the three-dimensional coordinates relative to the reference surface were obtained. However, it was observed that the precision decreased as the value of *z* approached regions where the displacement ratio dz/dx neared zero, indicating lower method sensitivity. Specifically, the sensitivity followed the relationship $dz/dx=-9.615\times 10^{-4}\cdot z_{0}+0.332$, meaning that, for lower values of $z_{0}$, the vertical displacement sensitivity decreased, increasing measurement uncertainty. In the context of bedridden patient posture detection, this loss of sensitivity may lead to reduced accuracy in height estimation near lower limb regions or when the projected pattern is partially occluded by bedding folds.

To validate the experimental results and optimize the experimental design, a simulation methodology was developed. Determining the positions from the images of the pattern captured by the camera requires establishing the relative positions and orientations of three reference systems: the laboratory system ($S_{0}$), which is defined by the wall and the table; the projector system ($S_{1}$), consisting of the laser and the diffractive element; and the camera reference system ($S_{2}$).

The relative positions and orientations of these reference systems were determined through a structured calibration process. The calibration began by projecting a structured light pattern onto a flat surface (wall) to establish a baseline. The orientation of the projector ($S_{1}$) was adjusted to align the projected lines with the pre-measured positions in the laboratory. The camera system ($S_{2}$) was then positioned so that the recorded image of the projected pattern closely matched the expected reference positions. To ensure consistency, the transformation parameters between $S_{0}$ and $S_{2}$ were iteratively refined by adjusting the camera’s tilt and distance until minimal deviation was observed between the measured and simulated positions of the projected lines.

This calibration process was critical for achieving accurate 3D reconstruction. Errors in positioning and orientation directly affect the accuracy of height estimations obtained from the structured light deformation. In particular, small misalignments can introduce errors in the extracted contour lines, impacting the detection of a person’s posture. The iterative refinement of the system mitigated these errors, ensuring the reliability of the method for its intended application in monitoring bedridden patients.

**Figure 8:**
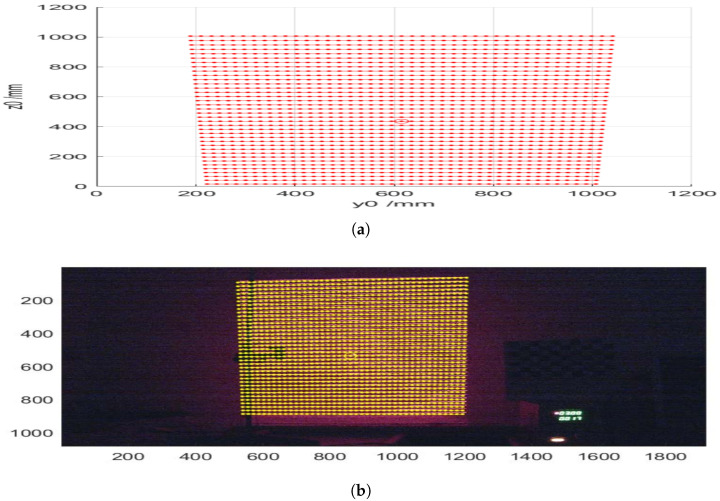
(**a**) Simulated lines. (**b**) Simulated lines over real projection.

The simulation of the projected pattern was essential for validating the calibration and experimental setup. This process involved generating a stripe pattern according to the specifications of the diffractive element used (Figure 8a). The next step was to compute the intersection of the projected stripes with the wall plane and to fine tune the projector’s orientation so that the detected line positions matched the laboratory measurements. Finally, the projected lines’ detected positions in the camera image were extracted and superimposed onto the real image for validation (Figure 8b). This step ensured that the spatial relationships between the reference systems were correctly established, minimizing errors in the final posture detection process.

Once the simulation is calibrated, it can be used for various applications. For instance, to analyze the vertical displacement of the stripes ($z_{0}$ axis) caused by a surface movement in the direction normal to the wall ($z_{0}$ axis), it is possible to simulate the stripes generated by two parallel planes and to calculate the ratio dz/dx, which varies with the height $z_{0}$.

**Figure 9:**
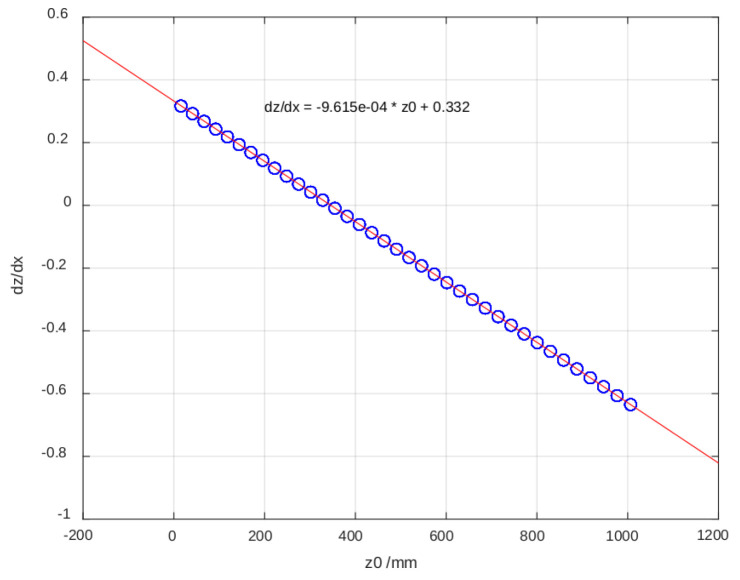
The dz/dx ratio that the sensitivity of the method for estimating the heights on the images of the lines is based on.

The resulting Figure 9 is critical because the dz/dx ratio determines the sensitivity of the method for estimating heights based on the images of the lines. When dz/dx approaches zero, the sensitivity decreases significantly, leading to larger errors in the estimations.

**Figure 10:**
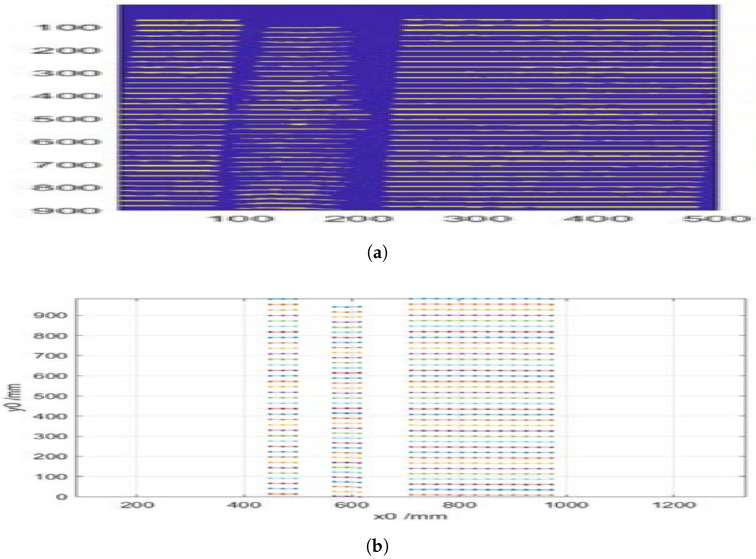
(**a**) Binarized image of the lines projected onto the cylinder. (**b**) Identified positions of the projected pattern lines onto the cylinder.

To determine the height on non-flat surfaces, the same procedure used to process the reference image was applied, obtaining the black-and-white version and the corresponding points of the lines. In the case of a cylindrical tube (Figure 10a,b), up to 39 lines were identified on its surface, which was 2 more than on the wall. This increase in the number of detected lines was due to the curvature of the cylinder, which causes a denser distribution of the projected pattern along the curved surface.

To establish the correspondence between the projected lines on different surfaces, a reference pattern was first captured on a flat wall to serve as a baseline. The known spacing of the lines in this reference allowed for tracking how the pattern deformed when projected onto the cylindrical surface. A linear relationship between the line number and height $z_{0}$ was established, following $z_{0}=-27.093\times \ln+1007.402$, where ln represents the line index. This equation, derived from the calibration process, enabled a precise mapping between the projected lines on different geometries.

However, shadowed areas were found at both ends of the cylinder, where no lines were detected due to occlusions. To handle these interruptions, the missing line segments were interpolated based on the neighboring detected points. This interpolation process helped maintain consistency in height estimation while mitigating errors introduced by shadows. The approach ensured that even in cases where partial occlusions occurred, the structured light system remained effective in extracting 3D surface information.

**Figure 11:**
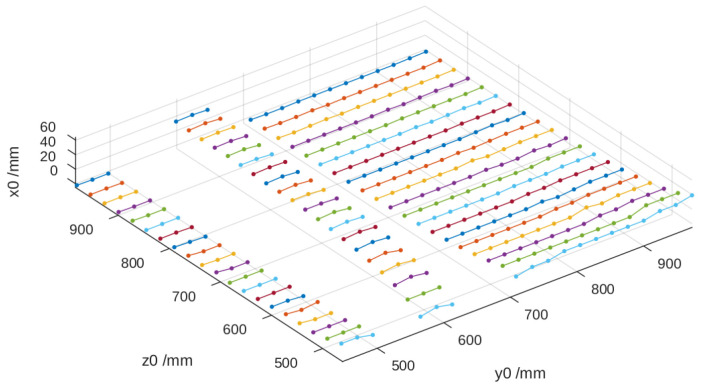
Height of the cylinder calculated from the reference image.

Using the vertical displacement of each line dz and the corresponding value of dz/dx, the height relative to the reference surface was calculated, thereby obtaining the three spatial coordinates. In Figure 11, it can be observed that the height values x0 deteriorate when z0 decreases as dz/dx tends to approach zero, which affects the method’s precision.

**Figure 12:**
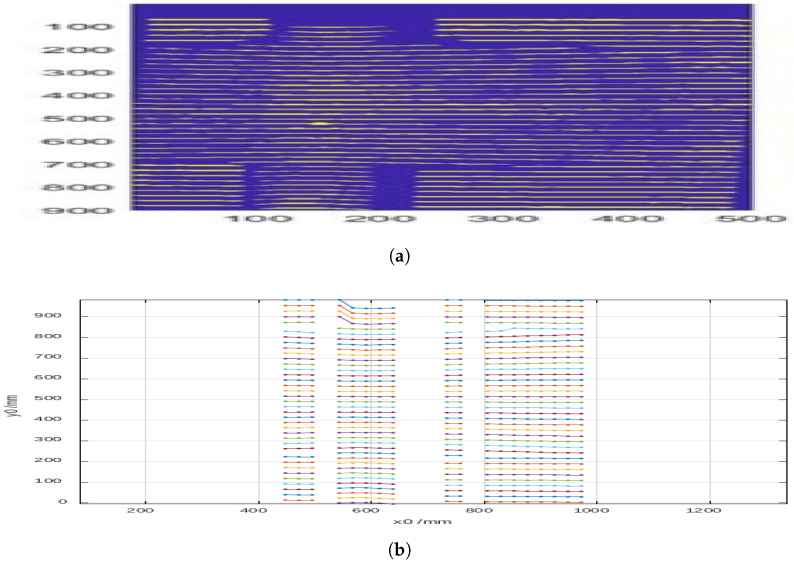
(**a**) A binarized image of the lines projected onto the cylinder under a sheet of paper. (**b**) The identified positions of the projected pattern lines onto the cylinder under a sheet of paper.

**Figure 13:**
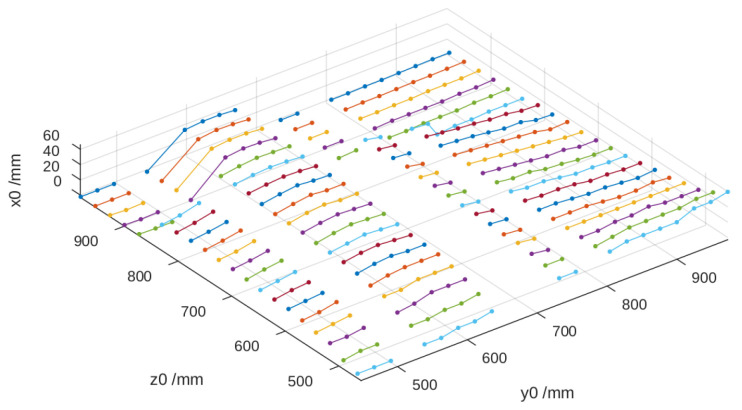
Height of the cylinder under a sheet of paper as calculated from the reference image.

In the case of a cylinder covered with a sheet of paper (Figure 12a,b and Figure 13), the results were similar, with the particularity that certain lines disappeared due to the characteristics of the surface.

The results obtained in the laboratory tests demonstrate that the system is capable of acquiring three-dimensional information using a single camera and diffractive patterns. However, limitations were identified related to the method’s sensitivity and the relative configuration of the elements. The primary issue affecting sensitivity is the decrease in measurement accuracy when the displacement ratio dz/dx approaches zero, particularly for lower values of $z_{0}$. The sensitivity follows the relationship $dz/dx=-9.615\times 10^{-4}\cdot z_{0}+0.332$, meaning that, for $z_{0}<200$ mm, the measurement uncertainty significantly increases due to the low variation in displacement.

Additionally, the relative configuration of the elements introduced errors due to the projection angle and occlusions in certain regions of the measured surface. The experimental data indicate that misalignment of the camera and laser by even $1.5^{\circ }$ led to a deviation of up to 5 mm in height estimation. Furthermore, the occlusions caused by folds in the blanket and the curvature of the body resulted in missing data points, requiring interpolation techniques that introduce additional uncertainty.

These limitations affect the final application by reducing the accuracy of the 3D surface reconstruction, particularly in areas where the patient’s body creates significant occlusions or where the system operates at lower height ranges. As a mitigation strategy, adjustments in the projector–camera positioning and the implementation of an adaptive filtering approach were explored to enhance the robustness of the measurement process.

#### 3.3.2. Real-World Testing

Once the system’s feasibility was demonstrated in the laboratory, it was tested in a real environment to further refine and validate its performance. With the intention of verifying whether we could improve measurement accuracy and increase the number of data points, we replaced the DOE with a parallel line pattern with one featuring a grid pattern.

**Figure 14:**
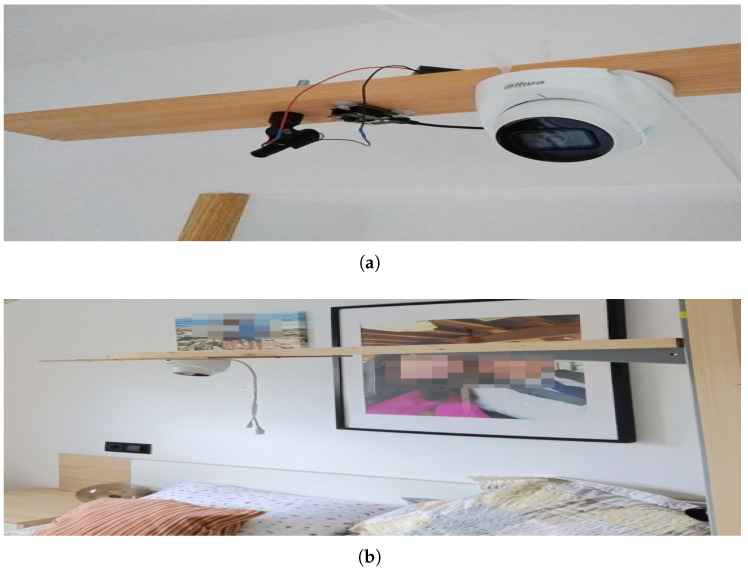
(**a**,**b**) The laser plus camera assembly configuration.

The experimental setup initially involved positioning the camera and laser assembly at a height of approximately 2 m, which was situated about 1 m from the foot of the bed. This configuration, as illustrated in Figure 14a,b, was angled at roughly 45° to maximize coverage of the bed surface. However, this arrangement presented several significant challenges that necessitated further adjustments to the experimental design.

The main issue encountered was the distortion introduced by the oblique projection angle, which caused a non-uniform distribution of the structured light pattern across the bed surface. This resulted in variations in the spacing between projected lines, leading to decreased accuracy when extracting the patient’s body profile. Additionally, due to the perspective effects, the areas closer to the camera exhibited higher resolution, while regions farther from the camera suffered from reduced measurement precision.

To address these challenges, the experimental setup was modified by repositioning the camera directly above the bed. This adjustment ensured a more uniform projection of the structured light pattern, minimized distortions, and improved the overall reliability of the system in detecting posture-related features.

**Figure 15:**
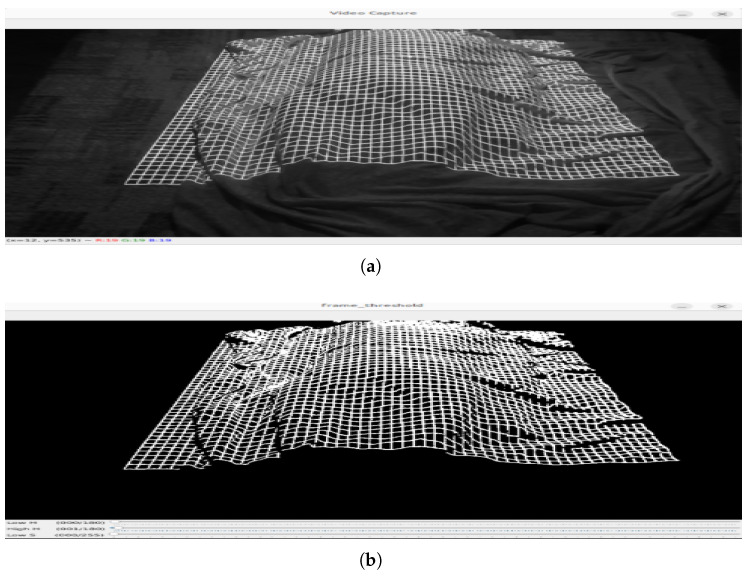
(**a**) Capture of an image using the DOE with a grid pattern. (**b**) Same image after applying filtering by HSV color model values to remove the background.

One of the main issues identified was the excessive density of the projected lines near the head of the bed. As shown in Figure 15a,b, the lines in this area were too close together, making precise detection difficult due to the relative position of the bed with respect to the laser assembly. This clustering compromised the system’s ability to distinguish individual projections, potentially affecting the accuracy of measurements in this area. To address this issue, the laser was kept in its initial position but the camera was relocated directly above the bed (Figure 14b). Initially, the camera and laser assembly were positioned approximately 2 m high and 1 m away from the foot of the bed, angled at roughly 45° to maximize coverage. However, this oblique perspective introduced distortions in the structured light projection, affecting the accuracy of the reconstructed surface.

**Figure 16:**
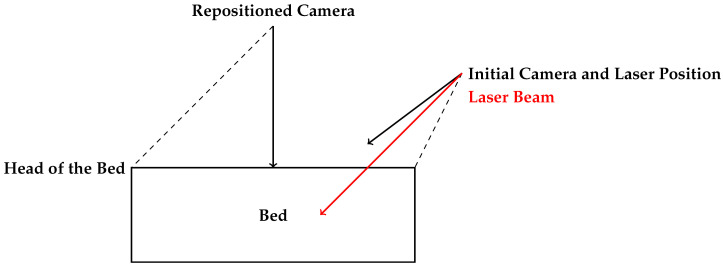
Schematic representation of the initial and adjusted experimental setups.

To mitigate these issues, the camera was repositioned directly above the bed, ensuring a more uniform and distortion-free projection of the structured light pattern. Since the available photographs do not fully illustrate the spatial relationship between the bed and the devices, a schematic diagram has been included (Figure 16) to clarify their relative positioning. This representation provides a clearer understanding of the experimental setup and the modifications made to optimize the system’s performance.

**Figure 17:**
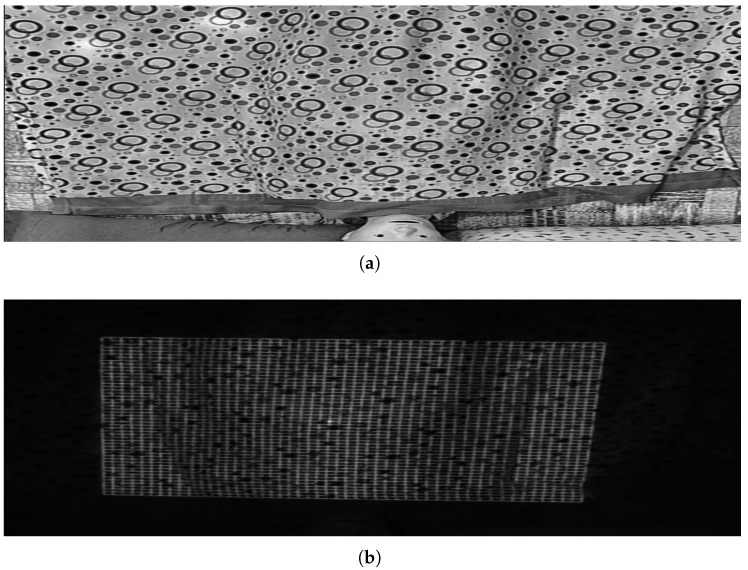
(**a**) Sheet with problematic colors. (**b**) Laser projection over the sheet with problematic colors.

Another challenge that emerged during the experiments was related to the reflective properties of different bed linen colors. Certain hues proved less effective at reflecting the laser light, resulting in poor visibility of the projected pattern, as shown in Figure 17a,b. However, this issue is unlikely to be a significant limitation in real-world applications as nursing homes, where the system is intended to be used, predominantly rely on light-colored, typically white, bed sheets. White linens are the standard in such environments because they facilitate the detection of stains and allow high-temperature washing with disinfectants without the risk of discoloration. Their use also simplifies laundry processes, reduces costs, and ensures consistent availability. Additionally, white conveys cleanliness, order, and professionalism, fostering a sense of trust and control in healthcare settings. Although some nursing homes may use light pastel tones, white remains the standard due to its practical and psychological benefits. Given these factors, variations in bed sheet color are unlikely to affect the system’s accuracy in its intended deployment environment.

The third significant problem arose from the interaction between the grid pattern and the test dummy. When the complete grid was projected onto the dummy, it became evident that the horizontal lines provided more valuable height information compared to the vertical lines. The presence of vertical lines introduced interference in the processing of the data, potentially compromising the accuracy of the height measurements. This observation led to a critical decision in the experimental approach.

In light of these findings, we concluded that continuing the experiments with the DOE projecting parallel lines would be the most effective path forward. This adjustment aims to capitalize on the more informative horizontal lines while eliminating the interference caused by the vertical components of the grid pattern.

**Figure 18:**
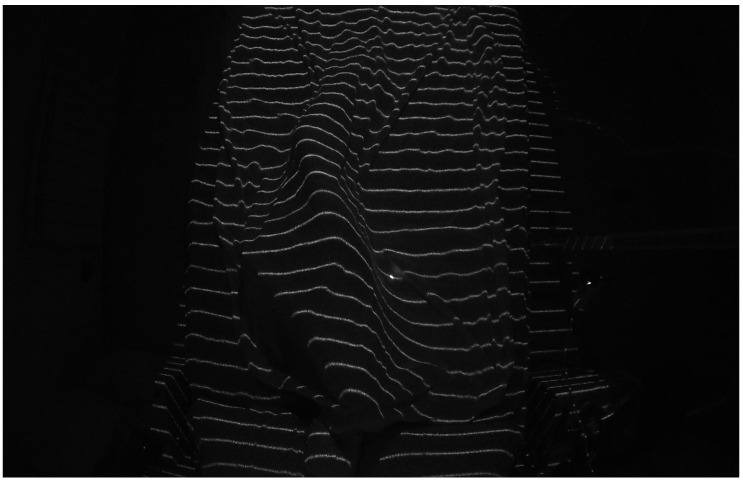
Segmented curves that are drawn by projecting the pattern of lines onto a body in bed.

When using the parallel lines DOE, we observed a more pronounced issue of interruptions in the projected lines compared to the laboratory tests conducted with the cylinder. This phenomenon was caused by multiple shadow zones created by the mannequin under the blanket, as shown in Figure 18. These interruptions made it impossible to accurately determine the correspondence between each projected line segment and its reference image on the empty bed, a correspondence that is crucial for precisely calculating the surface of the mannequin lying on the bed. We are currently working on solutions to address this issue, as previewed in Section 4.

A comprehensive analysis of the images obtained in our tests, as illustrated in Figure 18, revealed a significant simplification in the process of determining the patient’s position. Contrary to what might be expected, it is not necessary to reconstruct the entire body surface; instead, extracting the profile of the legs under the sheet is sufficient to determine whether the patient is lying on their left or right side. This simplification is based on the observation that the curvature of the leg profile provides a reliable indicator of lateral posture, reducing the computational complexity and avoiding the need for full-body surface reconstruction.

This profile is obtained by identifying the maximum points of each structured light line projected onto the body. The maximum points correspond to the highest elevations of the curves formed by the laser projection on the covered legs. To achieve this, the algorithm processes the structured light pattern by first segmenting the laser-projected lines within the region of interest (legs area). Each detected line is then analyzed to extract its local maximum, representing the peak deformation caused by the patient’s body. To ensure robustness, only lines meeting predefined geometric constraints—such as the minimum segment length and sufficient separation—are considered valid.

In the practical application of our structured light system, the process is further refined by fitting a second-degree polynomial (using the polyfit method of the Python NumPy library) to the set of the extracted maxima. This polynomial fitting allows for a smooth approximation of the leg profile while mitigating minor noise and projection irregularities. The concavity or convexity of the fitted curve is then evaluated, enabling a precise classification of whether the patient is lying on their left or right side.

However, this approach is specifically designed to classify lateral decubitus positions and does not provide information about whether the patient is lying in a supine position (on their back). Since the method does not analyze the upper body or head, it cannot distinguish between supine and lateral postures. This limitation is inherent to the simplification process, which prioritizes a minimally invasive and computationally efficient method over full-body posture classification. Future work may explore complementary techniques to extend the system’s capabilities to distinguish between additional postures if required for broader applications.

**Figure 19:**
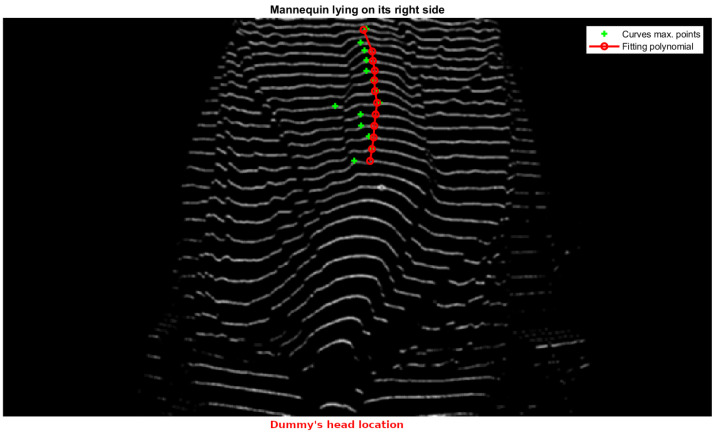
Dummy lying on its right side.

**Figure 20:**
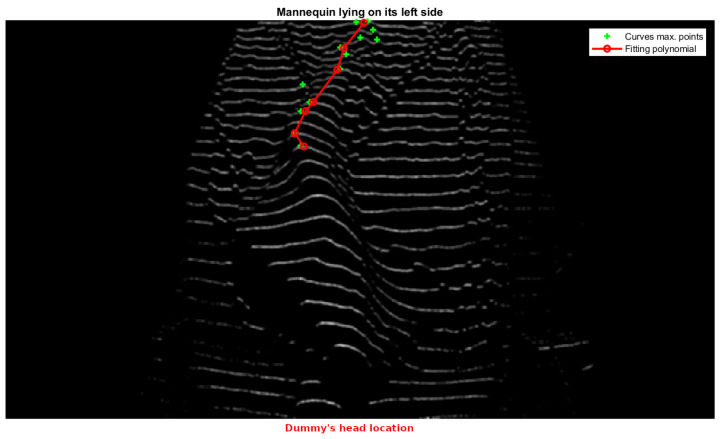
Dummy lying on its left side.

Figure 19 and Figure 20 illustrate this process, showing the mannequin lying on its right and left sides, respectively. Since the camera is positioned at the foot of the bed, the lower part of each image corresponds to the patient’s heels, while the upper part represents the position of the patient’s hips. This orientation is crucial for interpreting the results correctly.

In both images, green crosses mark the extracted maximum points from each projected line in the leg region, while the red curve represents the fitted polynomial derived from these maxima. As observed, the curvature of this polynomial—whether concave or convex—directly indicates the mannequin’s lateral posture, demonstrating the effectiveness of our method. Based on this orientation, Figure 19 corresponds to the mannequin lying on its right side, while Figure 20 corresponds to the mannequin lying on its left side.

## 4. Conclusions and Future Work

Current demographic trends reveal a notable increase in the elderly population, particularly in the group aged 80 and above. This situation demands innovative solutions in the field of healthcare and social support. In response, the Laboratory of Industrial Laser Applications at the University of A Coruña, in collaboration with Anta Norte and Raiola Residencial, has initiated the development of a cost-effective system to monitor bedridden individuals who require assistance in changing their position.

This project has investigated the feasibility of employing a laser-based structured light system to obtain a three-dimensional surface profile of a person in bed. Considering the high cost of cameras capable of capturing 3D surfaces and the complexity of creating datasets to train artificial intelligence models, an algorithmic approach was adopted. This method classifies the resting posture by analyzing the concavity or convexity of the leg profile curve.

The developed algorithm effectively determines the side on which a person is lying by calculating the concavity or convexity of the leg profile curve. This advancement was achieved by fitting a polynomial to the maxima of the curves projected onto the bed, representing significant progress in automating the monitoring process.

Regarding hardware, the structured light system, based on previous research and utilizing a laser with various diffractive optical elements, has demonstrated its effectiveness in generating detailed surface profiles, which are essential for patient assessment. The Dahua IPC-HDW2230T-AS-S2 camera (Dahua Technology, Hangzhou, China) proved ideal due to its high-resolution image capture capability and optimal performance in low-light conditions, which are crucial aspects for system accuracy. Specifically, the camera operates effectively in illumination levels as low as 0.005 lux in color mode and 0.0005 lux in black-and-white mode, allowing reliable detection in dimly lit environments such as hospital rooms at night or in nursing homes with subdued lighting. However, the system’s performance can be affected under high illumination conditions, such as direct sunlight or intense artificial lighting. The structured light projection relies on detecting the infrared laser pattern against the fabric of the bed linens, and excessive ambient light, especially if it includes infrared components, may reduce the contrast between the projected laser lines and the background, making it more challenging for the camera to distinguish them. To mitigate this, the system is designed to operate in controlled indoor environments where lighting conditions remain relatively stable. In future implementations, the inclusion of optical filters to block ambient infrared radiation or the use of more powerful laser sources may enhance robustness against varying illumination conditions.

Although laboratory tests were used to confirm the system’s precision under controlled conditions, the trials in real environments revealed significant challenges due to the deformation of the bedding when a patient is lying in the bed. One of the main limitations is that, by relying only on the maximum points extracted from the projected lines on the legs, we lose valuable information about volume changes in the patient’s body. Ideally, reconstructing the full surface would provide a more detailed assessment; however, with our current structured light system, this is not feasible. The primary obstacle is that the projected lines fragment into multiple segments when encountering the contours of the covered body, which is caused by shadows generated by the folds in the blankets and the shape of the patient’s body. This fragmentation disrupts the ability to establish a reliable correspondence between the detected segments and their respective original lines in the reference image, preventing an accurate reconstruction of the body’s surface.

**Figure 21:**
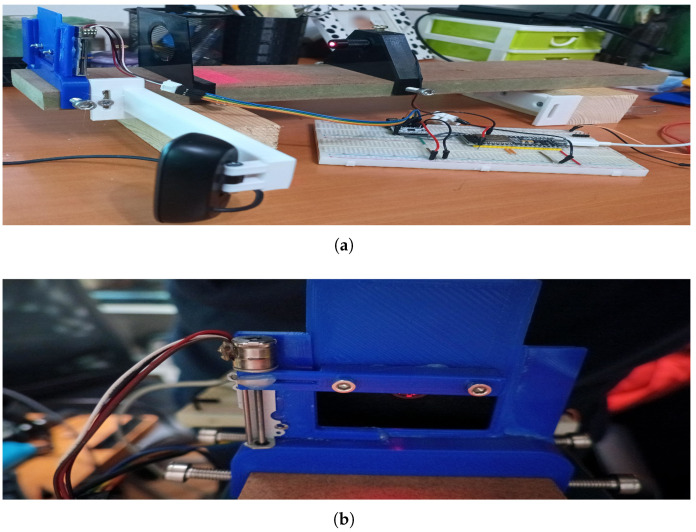
First prototype of the moving window mechanism.

To address this issue, we are currently developing a moving window mechanism (Figure 21a,b) that allows for sequential processing of the laser lines. This system is designed to expose each projected line one at a time, ensuring that the corresponding height values are computed before the next line is revealed. By processing the structured light pattern in a controlled sequence, the mechanism mitigates the challenge of establishing correspondence between fragmented segments of the projected lines and their original reference lines. This approach enables a more accurate reconstruction of the scanned surface, even in the presence of the shadow-induced discontinuities caused by the contours of the patient’s body and bedding folds.

Future research will focus on refining the moving window system. The goal is not only to determine the side on which a person is resting, but also to calculate their body surface to detect physical changes over time. Achieving this enhanced functionality may require additional equipment, such as a more precise actuator for the moving window mechanism or improved optical components to enhance measurement accuracy. However, maintaining the system’s low cost and simplicity remains a priority.

The proposed method was designed as a cost-effective alternative to existing solutions that rely on expensive depth cameras or pressure sensor arrays. Even with potential hardware refinements, the projected cost is expected to remain significantly lower than commercial 3D imaging systems, which often exceed several thousands of euros. Additionally, the structured light approach avoids the long-term degradation issues associated with pressure sensors and does not require the complex computational resources of machine-learning-based depth analysis. Future developments will carefully balance cost and performance to ensure that the system remains a practical and affordable solution for real-world applications.

While the system demonstrated promising results, the validation tests were conducted exclusively on a nursing mannequin. As a result, the algorithm has yet to be evaluated on a diverse range of leg contours. In real-world scenarios, variations in body morphology could influence the polynomial fitting process, potentially affecting classification accuracy. Therefore, future work will prioritize testing the algorithm on human subjects with different physical characteristics to ensure its robustness and adaptability.

In conclusion, this study demonstrates the viability of a low-cost structured light system for monitoring bedridden individuals, offering a scalable and effective solution for elderly care. The inter-institutional collaboration resulted in a prototype that addresses current limitations and lays the foundation for future advances in telecare technology. By refining the system and expanding its validation, this research has the potential to significantly improve the quality of life for older adults requiring continuous care.

## Figures and Tables

**Table 1:** KUROKESU C1 PRO X10 characteristics [19].

**Sensor**	Sony IMX290 1/2.8″ CMOS
**Resolution**	1920 × 1080
**Lens Type**	Motorized 10 × zoom lens
**Focal Length**	5.1–47 mm
**Aperture**	f/1.6–f/2.5
**Field of View**	**Wide**: 71°; **Tele**: 7.1°

**Table 2:** Dahua IPC-HDW2230T-AS-S2 characteristics [20].

**Sensor**	1/2.7″ 2 Megapixel progressive CMOS
**Resolution**	1920 × 1080
**Lens type**	Fixed-focal
**Focal Length**	2.8 mm; 3.6 mm
**Max. aperture**	**2.8 mm**: F1.6; **3.6 mm**: F1.6
**Field of view**	**2.8 mm**: Pan: 110°; Tilt: 59°; Diagonal: 132°. **3.6 mm**: Pan: 91°; Vertical: 48°; Diagonal: 109°

**Table 3:** List of component prices.

Component	Cost
Dahua IPC-HDW2230T-AS-S2	EUR 75.23
Picotronic DOE256-650-5-3	EUR 189.09
Picotronic DOE-DE-R284	EUR 54.89
**Total Cost**	EUR 316.3

## Data Availability

The data presented in this study are available on request from the corresponding author. The data are not publicly available due to ongoing research and privacy considerations.

## Appendix A. Algorithm

DeterminePatientPosition

Input: laser_curves // Curves drawn by laser with DOE parallel line pattern

// Step 1: Identify valid lines
valid_lines = []
for each curve in laser_curves:
if meetsGeometricConstraints(curve): // Check minimum length, separation, and position
valid_lines.append(curve)

// Step 2: Extract maximum points
max_points = []
for each line in valid_lines:
max_point = findMaximumPoint(line) // Identify the highest point in the curve
max_points.append(max_point)

// Step 3: Fit a second-degree polynomial
fitting_polynomial = polyfit(max_points, 2) // Quadratic fit to obtain smooth profile

// Step 4: Compute second derivative to determine concavity
second_derivative = calculateSecondDerivative(fitting_polynomial)

// Step 5: Determine position based on concavity/convexity
if second_derivative > 0:
position = “Left side” // Convex profile indicates left side
else:
position = “Right side” // Concave profile indicates right side

Output: position

-------------------------------------------------

Function meetsGeometricConstraints(curve)

Input: curve // List of (x, y) points of a projected laser line

// Define geometric constraints
min_length = 40
min_separation = 5
y_max_threshold = 188
x_range = (380, 500) // Valid X range for legs detection

// Calculate length of the curve
curve_length = sum( sqrt( diff(curve.x)^2 + diff(curve.y)^2 ) )

// Find global minimum y (highest point in image coordinates)
y_min = min(curve.y)
x_min = x value at y_min

// Check constraints
if curve_length > min_length AND
(x_min - min(curve.x) > min_separation) AND
(max(curve.x) - x_min > min_separation) AND
(x_min > x_range[0] AND x_min < x_range[1]) AND
(max(curve.y) - y_min > min_separation) AND
(y_min <= y_max_threshold):

return True // Curve is valid

return False // Curve does not meet constraints

-------------------------------------------------

Function findMaximumPoint(curve)

Input: curve // List of (x, y) points of a projected laser line

// Find the y-minimum (which represents the highest point due to image coordinate system)
y_min = min(curve.y)

// Get the corresponding x-coordinate for the minimum y
x_min = x value at y_min

return (x_min, y_min) // Maximum point of the curve

-------------------------------------------------

Function calculateSecondDerivative(polynomial)

Input: polynomial // Quadratic polynomial fitted to the maximum points

// A second-degree polynomial is in the form: ax^2 + bx + c
a, b, c = polynomial.coefficients // Extract coefficients

// The second derivative of ax^2 + bx + c is simply 2a
second_derivative = 2 * a

return second_derivative
